# Supplementary material for: The Systems Biology Research Tool: evolvable open-source software
Source: BMC Syst Biol. 2008 Jun 29;2:55. doi: 10.1186/1752-0509-2-55 (PMC2446383; doi:10.1186/1752-0509-2-55)
Supplement: Additional file 1 — SBRT Archive. An archive of the current version of the Systems Biology Research Tool. [file 1752-0509-2-55-S1.zip › sbrt-1.4.0/doc/developers_guide/api/sbrt/kernel/math/stat/comp/package-summary.html]

sbrt.kernel.math.stat.comp


|  |  |  |
| --- | --- | --- |
| **Overview** \| **Package** \| Class \| **Use** \| **Tree** \| **Deprecated** \| **Index** \| **Help** | |  |
| **PREV PACKAGE**   **NEXT PACKAGE** | **FRAMES**    **NO FRAMES**     **All Classes** |  |


---

## Package sbrt.kernel.math.stat.comp

Provides classes and interfaces for performing statistical computations.

**See:**
  
          **Description**

| **Interface Summary** | |
| --- | --- |
| **Correlator<V,T extends CorrResult>** | This interface is used to represent correlation statistic computers. |
| **CorrResult** | This interface is used to represent objects that store the results of a correlation computation. |

| **Class Summary** | |
| --- | --- |
| **KendallTau<V>** | This abstract class is a skeleton implementation of a Kendall's tau correlation statistic computer. |
| **KendallTauA<V>** | This class is used to compute Kendall's Tau_A_ correlation statistic. |
| **KendallTauB<V>** | This class is used to compute Kendall's Tau_B_ correlation statistic. |
| **MannWhitneyResults** | This class is used to store the results of a Mann-Whitney U test. |
| **MannWhitneyU** | This class is used to compute Mann-Whitney U statistics. |
| **TauResult** | This class is used to store the results of a correlation computation. |

## Package sbrt.kernel.math.stat.comp Description

Provides classes and interfaces for performing statistical computations.

---


|  |  |  |
| --- | --- | --- |
| **Overview** \| **Package** \| Class \| **Use** \| **Tree** \| **Deprecated** \| **Index** \| **Help** | |  |
| **PREV PACKAGE**   **NEXT PACKAGE** | **FRAMES**    **NO FRAMES**     **All Classes** |  |


---
